# The evolution of mental health related policies in China: A bibliometric analysis, 1987–2020

**DOI:** 10.3389/fpubh.2022.964248

**Published:** 2022-11-24

**Authors:** Haiyan Li, Qingyu Zhou, Hao Zhu, Peiwu Shi, Qunhong Shen, Zhaoyang Zhang, Zheng Chen, Chuan Pu, Lingzhong Xu, Zhi Hu, Anning Ma, Zhaohui Gong, Tianqiang Xu, Panshi Wang, Hua Wang, Chao Hao, Chengyue Li, Mo Hao

**Affiliations:** ^1^Research Institute of Health Development Strategies, Fudan University, Shanghai, China; ^2^Collaborative Innovation Center of Social Risks Governance in Health, Fudan University, Shanghai, China; ^3^Department of Health Policy and Management, School of Public Health, Fudan University, Shanghai, China; ^4^Zhejiang Academy of Medical Sciences, Hangzhou, Zhejiang, China; ^5^School of Public Policy and Management, Tsinghua University, Beijing, China; ^6^Project Supervision Center of National Health Commission of the China, Beijing, China; ^7^Department of Grassroots Public Health Management Group, Public Health Management Branch of Chinese Preventive Medicine Association, Shanghai, China; ^8^School of Public Health and Management, Chongqing Medical University, Chongqing, China; ^9^School of Public Health, Shandong University, Jinan, Shandong, China; ^10^School of Health Service Management, Anhui Medical University, Hefei, Anhui, China; ^11^School of Management, Weifang Medical University, Weifang, Shandong, China; ^12^Committee on Medicine and Health of Central Committee of China Zhi Gong Party, Beijing, China; ^13^Institute of Inspection and Supervision, Shanghai Municipal Health Commission, Shanghai, China; ^14^Shanghai Municipal Health Commission, Shanghai, China; ^15^Jiangsu Preventive Medicine Association, Nanjing, Jiangsu, China; ^16^Changzhou Center for Disease Control and Prevention, Changzhou, Jiangsu, China

**Keywords:** mental health, policy analysis, bibliometrics, policy evolution, China

## Abstract

**Background:**

Since 1987, the Chinese government has promoted public mental health by continuously implementing mental health related policies. This research attempts to reveal the distribution and characteristics of mental health related policies. In addition, it can help stakeholders evaluate whether the environment for policy implementation has improved and identify key points in the development of the overall mental health system.

**Methods:**

We used a bibliometric approach to analyze the evolution of mental health related policies in China from 1987 to 2020. A total of 239 mental health related policies were collected from Beida Fabao and official Internet websites of governmental departments. Co-wording, social networks, and citation analysis were applied to explore the evolutionary features of such policies.

**Results:**

The evolution of policy development showed that the number of mental health related policies in China has been increasing and their content has been enriched. Over time, mental health related policies not only gradually expanded its focus on common mental disorders, but also included an increasing number of keywords related to service provision, organization and administration. However, most policies were implemented independently by separate agencies and the number of policies jointly implemented by different agencies only accounted for 32.64% of all the policies implemented. The Ministry of Health (MOH) is at the core of the collaborative network associated with implementing mental health related policies in China.

**Conclusion:**

The environment associated with the implementation of mental health related policies in China is gradually improving. However, cross-sector collaboration among different agencies needs to be strengthened and financial support for related resources needs more attention. A clear division of responsibilities among various agencies and a sustainable financing mechanism are essential to the development and implementation of mental health related policies.

## Introduction

Mental disorders are currently considered a major public health and social issues that significantly affect economic and social development (1). The WHO Special Initiative for Mental Health [2019–2023] stated that mental health must be an integral part of Universal Health Coverage (2). However, existing study of mental health literacy in China has shown that public recognition of mental disorders is still low (3). As of 2019, the prevalence and lifetime prevalence of mental disorders in China was estimated at 9.3 and 16.6%, respectively, with the disease burden accounting for 7.4% of the total disease burden (4). It is predicted that depression will become the mental disorder with the highest disease burden by 2030 in China (5).

Mental health related policies are considered to play an important role in the practice of preventing and reducing mental disorders (6). Over recent years, the Chinese government has formulated and implemented a series of different mental health related policies. Meanwhile, the importance of government leadership and the associated cross-sector organizations as well as their management is continuously emphasized. As clearly presented in the “China Mental Health Work Plan (2002–2010)” (7) and the “National Mental Health Work Plan (2015–2020)” (8), a comprehensive management and service mechanism should be formed. Overall, the implementation of mental health related policies in China has been improving over time.

In recent decades, research on mental health policy mainly focused on evaluating policy effects and analyzing influencing factors. Regarding policy evaluation, scholars have mainly conducted studies based on the mental health policy evaluation framework. For example, Faydi et al. reported the results of an assessment of Ghana, South Africa, Uganda, and Zambia's mental health policies using the WHO Mental Health Policy Checklist. The conclusions pointed to gaps that could impact on the policies' effect on countries' mental health systems, including inadequate political support and a lack of financial specificity (9). Wong et al. (10) evaluated the content of mental health related policies in terms of financing, coordination, and human resources from 2002 to 2012 drawing on the framework of mental health policy developed by the WHO. In terms of policy influencing factors, it was influenced by various aspects. Gopalkrishnan et al. explored some critical considerations at the intersection of cultural diversity and mental health such as establishing active resource collaboration with the community (11). Stangl et al. (12) identified health stigma and discrimination as essential factors affecting the implementation of mental health policies (12). Liang et al. (13) examined the challenges to integrating mental health services into China's general healthcare system, including: accurately estimating mental health needs, integrating mental and physical healthcare, and increasing workforce development and training (13).

Moreover, an increasing number of studies on the evolution of mental health related policies have been conducted in different regions or countries (14). For example, Bilir et al. (15) offered a brief history and the evolution of mental health policy in Turkey through a qualitative analysis (15). Draper et al. (16) analyzed the content and development process of mental health policy in South Africa (16). In China, Ma et al. (17) conducted a systematic review of mental health related policies from 2000 to 2009. Chen et al. (18) analyzed the characteristics of mental health related policies from 2009 to 2019. We found that most of these studies have adopted a qualitative approach to analysis and the time span covered by existing studies is 10 years, which is relatively brief. These studies have prompted us to comprehensively conduct quantitative analyses over a longer time span, which is essential for grasping the trends of such policies.

Currently, bibliometric analysis has become a widely used method for policy research, with the advantages of systematically revealing changes in policy content and analyzing the structure and evolution of policy systems (19, 20). It has been applied to the analysis of disaster policy (21), rural informatization policy (22), and nuclear energy policy (23). In the field of health policy, Fusco et al. (24) analyzed co-production in health policy and management through a comprehensive bibliometric. It concluded that there was a shortage regarding co-delivery and co-management, as well as the evaluation of their real impacts on providers and patients (24). Wu et al. (25) revealed and characterized the evolution of patterns of China's policy against COVID-19 from the perspective of policy making by using bibliometric methods (25). Nan et al. (26) used a bibliometric analysis method to probe into the evolution of Chinese aging policies from 1978 to 2019 (26). However, few studies have analyzed mental health related policies from a quantitative perspective.

To fill this research gap, we conducted a systematic analysis of mental health related policies by using bibliometric analysis. This study aims to analyze the distribution and characteristics of mental health related policies in terms of changes in policy content and cooperative networks of policy issuing agencies. To achieve this, we need to address the following three questions: (i) What are the distribution characteristics of mental health related policies in China since 1987? (ii) What are the emphases and changes in the content of mental health related policies at different stages of development? (iii) How are governmental departments and their collaborators involved in mental health related policies distributed? What are the decision-making networks and their relationships? The year 1987 was chosen as the starting point for this analysis because the WHO, in cooperation with the Ministry of Health (MOH), the Ministry of Public Security (MOP), and the Ministry of Civil Affairs (MOC), conducted the first formal discussion on mental health legislation in China in 1987, which marked the formal beginning of the mental health related policies system in China (10).

These three specific questions can help to accurately identify stage focus and development trends on mental health related policies. As the topics of mental health related policies keep shifting, a clear understanding of the policy evolution dynamics is essential for stakeholders. For practitioners, analyzing the collaboration structures can facilitate a better understanding of the distribution of responsibility in different government departments in policy design. For academics, it can enrich policy research in the field of mental health.

The rest of this paper was organized as follows: the methods section accounts for study design, data collection, study methods and statistical analysis. Next, the results section was analyzed regarding time distribution characteristics, emphasis and change of policy content, and the policy-issuing agencies. Finally, based on these results, we discussed and concluded them with the previous studies.

## Methods

### Study design

**Figure 1:**
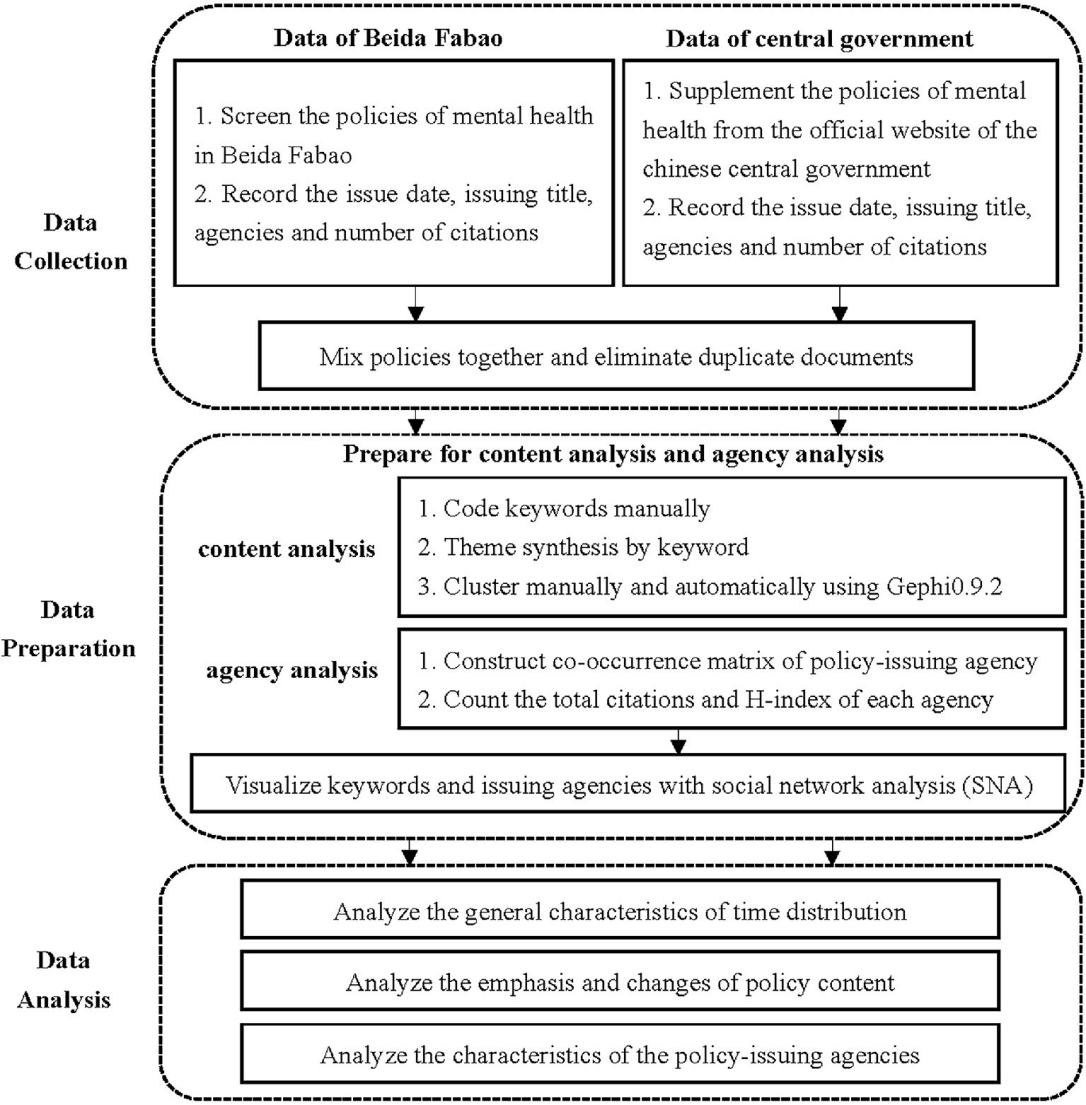
Research framework.

A bibliometric approach was applied to analyze mental health related policies issued in China from 1987 to 2020. The number of policies at different stages was counted, and the changes in policy content and collaborative networks of mental health issuing agencies were analyzed by co-word analysis and social network analysis. The process was divided into three main steps: data collection, data preparation, and data analysis. The steps of the study are shown in Figure 1.

### Data collection

The data were collected from “Beida Fabao” (http://www.pkulaw.cn), which is the most authoritative and comprehensive full-text policy database in China. We further searched the official websites of the government as supplementary databases for data validation, including the MOH, the Ministry of Finance (MOF), and the Ministry of Education (MOE), for normative documents related to mental health. The keywords used were “mental health,” “mental wellbeing,” “mental disorders,” and “mental illness.” The time span was from January 1987 to December 2020. A total of 391 policy documents were collected.

To ensure the accuracy of the data, a range of criteria was used for filtering the collected policy documents. The criteria were as follows: (a) The duplicated policies were removed; (b) Combined with the characteristics of keywords in mental health related policies, policy documents that had no guidance value and practical content or were not closely related to mental health were removed; (c) The policy types included laws, regulations, notices, announcements, measures, opinions, outlines, methods, and decisions, among others, while catalogs, supervision and inspection documents, and industry standards, among others, were excluded. A total of 239 policy documents were finally obtained. The date, title, issuing agency, and citation number of each policy were recorded individually.

Team members read each policy document to develop a preliminary understanding of policy contents before formal coding. After unified training and trial coding, two researchers in our team coded 239 policy documents separately. They were asked to give keywords according to the policy contents. After coding, synonyms with these similar terms were merged into the same keywords for each policy. Furthermore, similar keywords coded by two researchers were merged. Inconsistent codes were discussed and determined by the two researchers. The coding consistency coefficient was an acceptable value of 86.2%. The number of keywords for each policy was, on average, two to ten.

### Study methods

A thematic synthesis has often been applied to health policy and systematic evaluation research (27, 28). Based on the descriptive themes and action areas suggested by the WHO (6), which include organization and management, service provision, resources, and the interaction between subjects and objects of policy service (29), the content of mental health related policies was conducted through cluster analysis in the following four aspects: (a) Service objects: referring to the target groups of the mental health system, including people with mental disorders and the general population; (b) Service provision: including propaganda and education, psychological therapy, community rehabilitation, psychological intervention, and the provision of essential drugs; (c) Organization and administration: including how mental health services are organized, monitoring and evaluation of policy implementation, coordination within mental health systems and among all levels of governments, designation of agencies' responsibilities, and collaboration across sectors; and (d) Resources: including human resources, financial support, facility construction, information resources, and social welfare required for mental health services.

Co-word analysis is a method that was first proposed by Callon et al. (30), which assumes that keywords can be understood as an adequate description of a research theme (31). The words mentioned with high frequency were used as the basis, and the relationship between a set of keywords was established by counting the number of occurrences in the same text (32).

Social network analysis is a common method for analyzing the relational structure and properties of social networks and measuring the degree of association between them (33). The policy-issuing agencies were regarded as actors in the network, and the collaborative issuing of documents by the two agencies was considered as links to map the social network; thus, the connection between them can be analyzed.

### Statistical analysis

The statistical analysis was divided into three main steps: (i) The trend of mental health related policies issuing documents was described using Excel 2019 (Microsoft, Redmond, WA, USA); (ii) The co-occurrence matrix of keywords and issuing agencies was constructed using Bibexcel 1.0.0.0 (Umea University, The Information Research Group, Inforsk, Sweden) and the keywords were automatically and manually clustered by the topic synthesis; and (iii) Visualization analysis was performed using social network analysis (SNA) and social network graphs of keywords and issuing agencies were drawn using Gephi 0.9.2. By counting the citation frequency of policies issued by each agency, we calculated the H-index (34) corresponding to the issuing agency. An agency has an index of H if H of its N policy documents have at least H citations each and the other (N-H) policy documents have ≤ H citations each. The higher the H-index, the greater the administrative influence of the policy-issuing agency.

## Results

### Characteristics of time distribution

**Figure 2:**
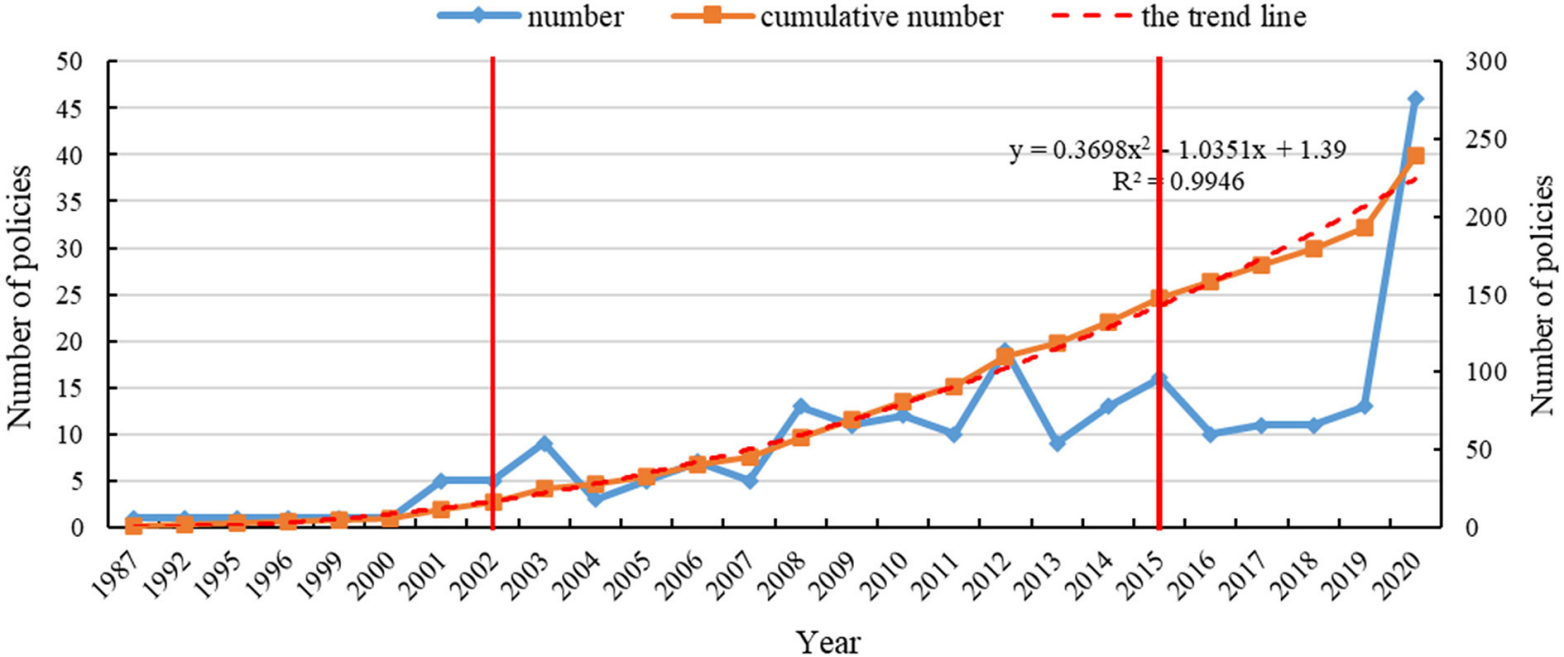
Time distribution of mental health related policies, 1987–2020.

Figure 2 shows the distribution of the number of mental health related policies issued per year from 1987 to 2020, dividing the development of mental health into three stages with the two major plans issued by China as time nodes.

From 1987 to 2001, the number of mental health related policies issued remained stable. From 2002 to 2014, China attached increased importance to mental health and the number of issued documents generally showed an increasing trend. In 2002, the MOH, the MOP, and the China Disabled Persons' Federation (CDPF) jointly issued “the China Mental Health Work Plan [2002-2010],” which comprised the first long-term plan for mental health. The SARS epidemic in 2003 captivated the attention of the entire society regarding the importance of public health. As a result, the disease prevention and control system, including the mental health system, was strengthened. The number of policies reached a peak, with nine mental health related policies issued in 2003. After 2003, there was a brief decline in the number of policies, but the overall trend was upward. In 2012, the number of mental health related policies issued was 19. This year also saw the enactment of the first law in mental health (35), thus legally regulating the field. Compared to the previous stage, the number of mental health related policies fluctuated significantly from 2015 to 2020. The year 2015 was a key point for the steady development of mental health work in China. The MOH, the National Development and Reform Commission (NDRC), and eight other agencies jointly issued “the National Mental Health Work Plan [2015-2020],” which provided an overall plan for mental health from 2015 to 2020. After the outbreak of COVID-19 at the end of 2019, its impact on the isolated population and people with mental disorders could require long-term attention. The number of policies issued that were related to mental health was 46 in 2020, reaching its peak.

In Figure 2, the cumulative number of policies line (the solid orange line) and its trend line (the red dotted line) were formulated to better portray the trends of releasing policy. The polynomial function was selected to fit the line according to the trend of releasing policy. The cumulative number of policies line can be perfectly fitted by the curve Y = 0.3698X^2^ −1.0351X + 1.39, where the value *R*^2^ equaled 0.9946. Before 2017, the solid orange line was located slightly above the red line or they overlapped each other. However, the solid orange line was located below the red dotted line from 2017 to 2019, indicating that the government and its departments have attached less importance to the field of mental health in China. The number of policies issued has decreased. After 2019, the number of policies increases significantly again, indicating the great importance attached to mental health, which may be related to the COVID-19 outbreak that brought more serious challenges to the field of mental health.

### Emphasis and change of policy content

**Figure 3:**
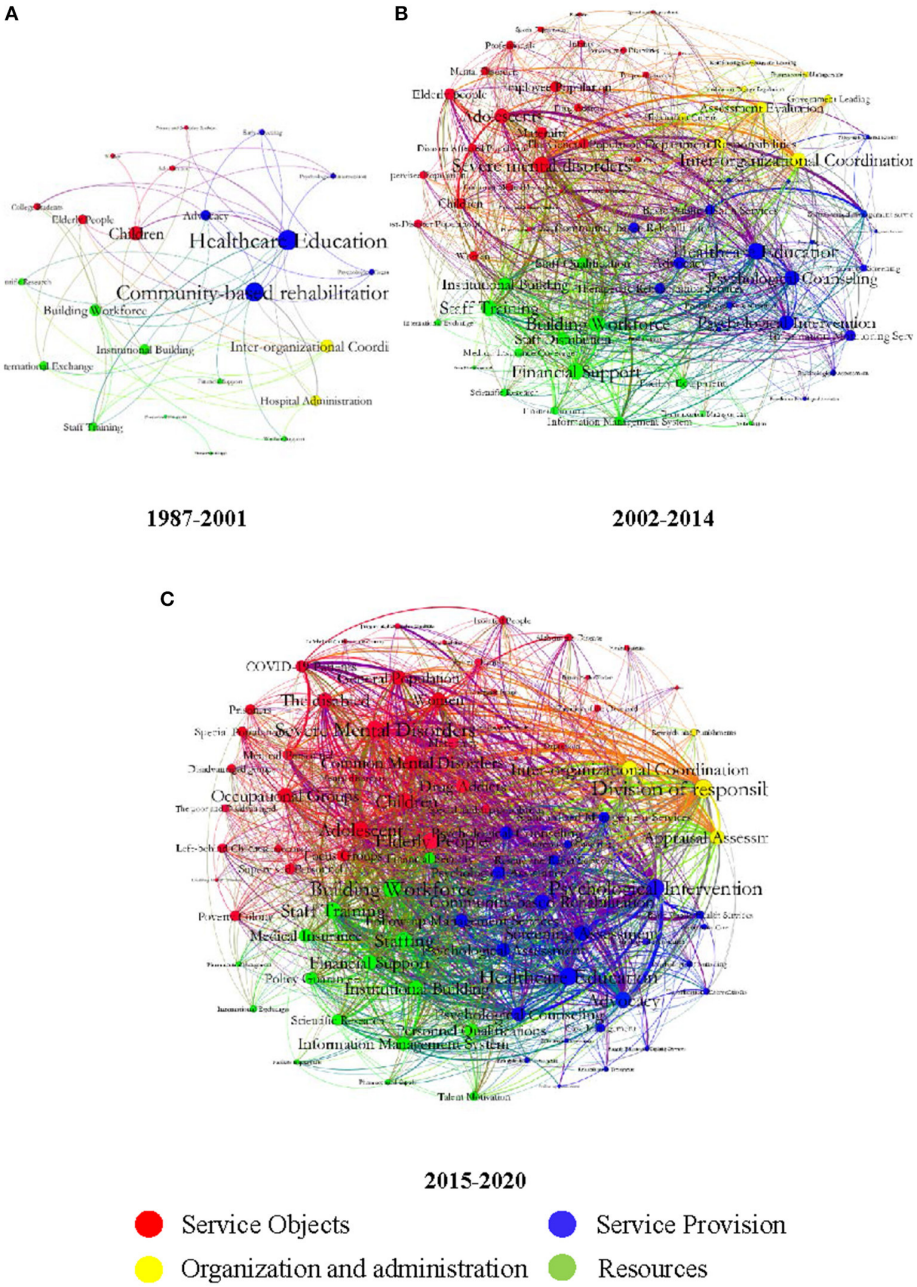
Keywords network of China's mental health related policies. **(A)** In the stage from 1987 to 2001. **(B)** In the stage from 2002 to 2014. **(C)** In the stage from 2015 to 2020. Policy-related keywords are divided into four categories with four colored dots.

Based on the four domains of mental health related policies' content clustering, the results of the extracted keyword clustering were shown in Figure 3.

China's mental health related policies were in the initial development stage from 1987 to 2001. The service objects involved children, teenagers, and college student groups. The service provisions were mainly measures, such as mental health/healthcare education, community-based rehabilitation and publicity, and popularization services. In terms of organization and management, the coordination of organizations and the management of psychiatric hospitals were mainly mentioned (Figure 3A, 1987–2001).

From 2002 to 2014, compared with the previous stage, China implemented the National Basic Public Health Service Project in 2009, which included management services for severe mental disorders. Compared with other objects, severe mental disorders received the most attention and were mentioned most frequently. Notably, during this period, the SARS outbreak and Wenchuan earthquakes occurred in 2003 and 2008. The survivors and occupational groups, which included medical staff and rescuers, were widely considered as those who were affected by these major public health emergencies. In terms of service provision, measures such as psychological counseling, psychological assessment, and information monitoring, increased during this period. Correspondingly, financial support increased in terms of resources, but the attention paid to financial support remained low. Management measures such as the division of responsibilities and assessment and evaluation, have been added to organization and management categories (Figure 3B, 2002–2014).

The development of mental health related policies in China further accelerated from 2015 to 2020. As shown in the overall keyword network diagram (Figure 3C, 2015–2020), there was an expansion in the four domains of service objects, service provision, organizational and management measures, and resources. At this stage, severe mental disorders were still the main service objects. However, compared with the previous stage, the service objects were more specialized, including groups such as left-behind children and primary health workers. Further, the attention paid to common mental disorders increased. After the outbreak of COVID-19 at the end of 2019, the service objects of the policy were mainly COVID-19 patients, susceptible people, and isolated people. In terms of service provision, new measures, such as information management and classification interventions were added. Regarding resources, the attention paid to financial support for mental health needed to be further improved. In terms of organization and management, the keywords of the division of responsibilities received the highest attention.

### Characteristics of the policy-issuing agencies

The connections between mental health related policy-making agencies can be discussed, in general, by determining the number of policies issued separately or jointly. Since 1987, 50 agencies have been involved in issuing mental health related policies.

**Figure 4:**
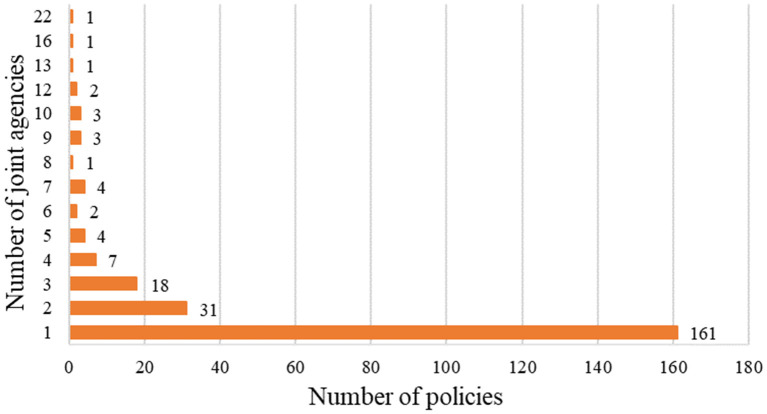
Distribution of agencies co-operation.

**Figure 5:**
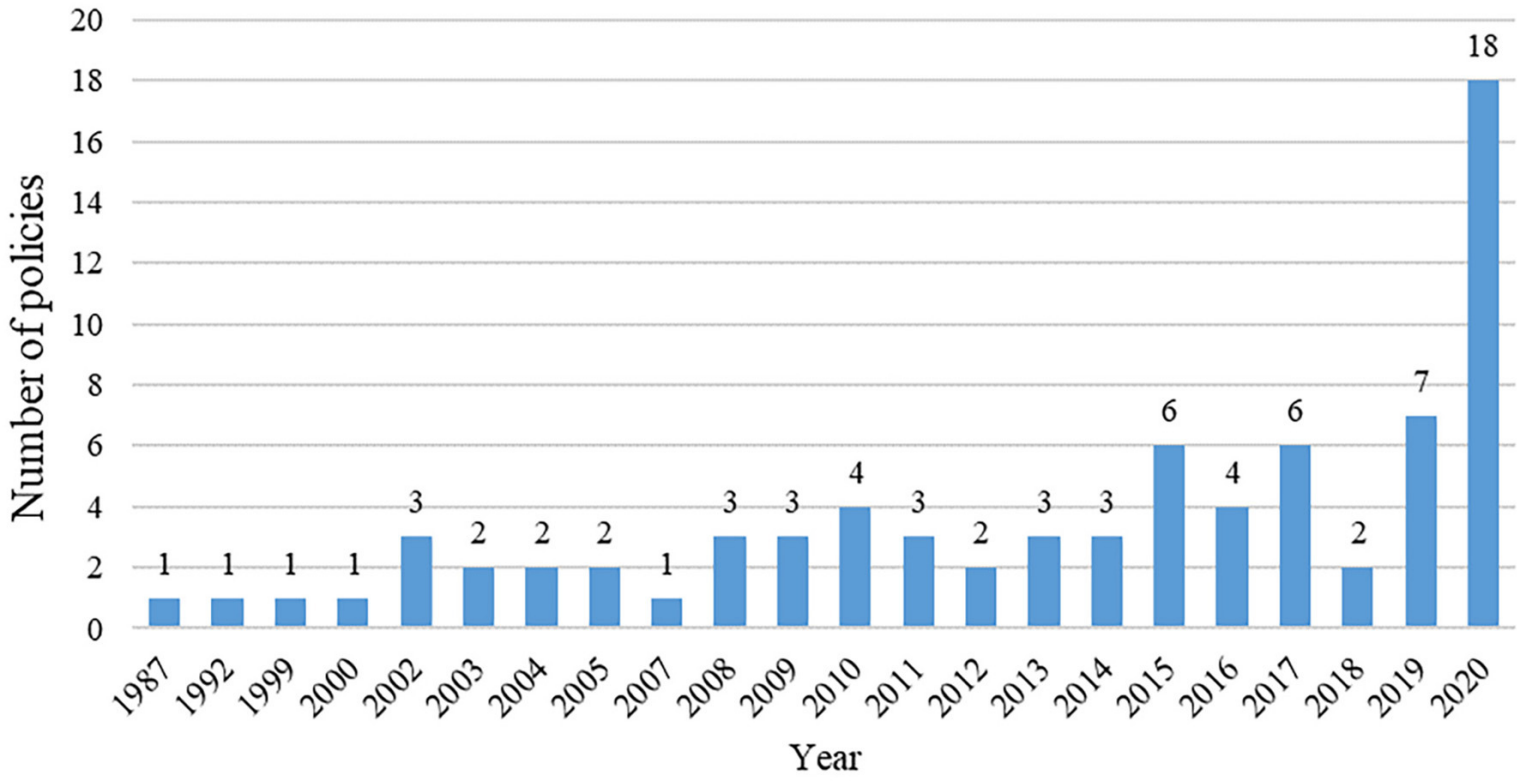
The number of policies issued jointly per year.

Generally, most of the policies were issued by separate agencies, with relatively few policies issued jointly by agencies (Figure 4). According to our statistics on the agencies issuing mental health related policies, 161 policies were issued by a single agency (accounting for 67.36% of the total) and 78 policies were issued jointly (32.64%). The policy that was jointly issued by the largest number of agencies was “the Guidelines Regarding the Strengthening in Mental Health Services (36).” It was issued in 2016 by 22 agencies (including the MOH, the NDRC, the MOF, the MOE, the MOP, and the MOC) as the first macro-guidance approach for strengthening mental health services in China. This was a typical case of collaboration among various agencies. In addition, we found that the number of jointly issued policies generally showed an increasing trend (Figure 5). Approximately 4.64 policies on average were issued each year from 2007 to 2020.

Statistical analysis was conducted on the citation frequency of policies issued by each agency, and the H-index was calculated (Table 1; only those with a total of five or more policies issued by agencies were listed here). According to the H-index, it was found that the main agencies on mental health related policies were the MOH, the MOF, the MOC, the MOE, and the State Council of China (SC). The MOH issued the highest number of mental health related policies. Since 1987, it has issued 132 policies, accounting for 55.23% of all such policies. Thus, the MOH plays a significant role and is a leading agency with a high participation frequency in mental health related policies.

**Table 1:** The number of policies and H-index of mental health related policies making agencies in China, 1987–2020.

**Issuing agency**	**Total number issued**	**Issued separately**	**Issued jointly**	**H-index**
The state council of China (SC)	33	29	4	24
Ministry of health of the people's republic of China (MOH)	132	65	67	21
Ministry of education of China (MOE)	52	30	22	20
Ministry of finance of China (MOF)	35	0	35	15
Ministry of civil affairs of the people's republic of China (MOC)	39	4	34	11
National administration of traditional Chinese Medicine (NATCM)	21	0	21	9
Ministry of human resources and social security of China (MOHRSS)	12	0	12	7
Ministry of public security of China (MOP)	12	0	12	7
The central committee of the communist young league (CCYL)	10	0	10	7
China disabled persons' federation (CDPF)	17	2	15	6
National development and reform commission (NDRC)	11	0	11	6
Ministry of justice (MOJ)	12	3	9	6
All-China women's federation (ACWF)	9	0	9	6
Publicity department of the communist party of China (CCPPD)	8	0	8	5
National radio and television administration (NRTA)	5	0	5	4
National working commission on aging (NWCA)	5	0	5	4
Comprehensive group of joint prevention and control mechanisms (GJPC)	11	11	0	4
The national people's congress of the people's republic of China (NPCPRC)	7	7	0	0

**Figure 6:**
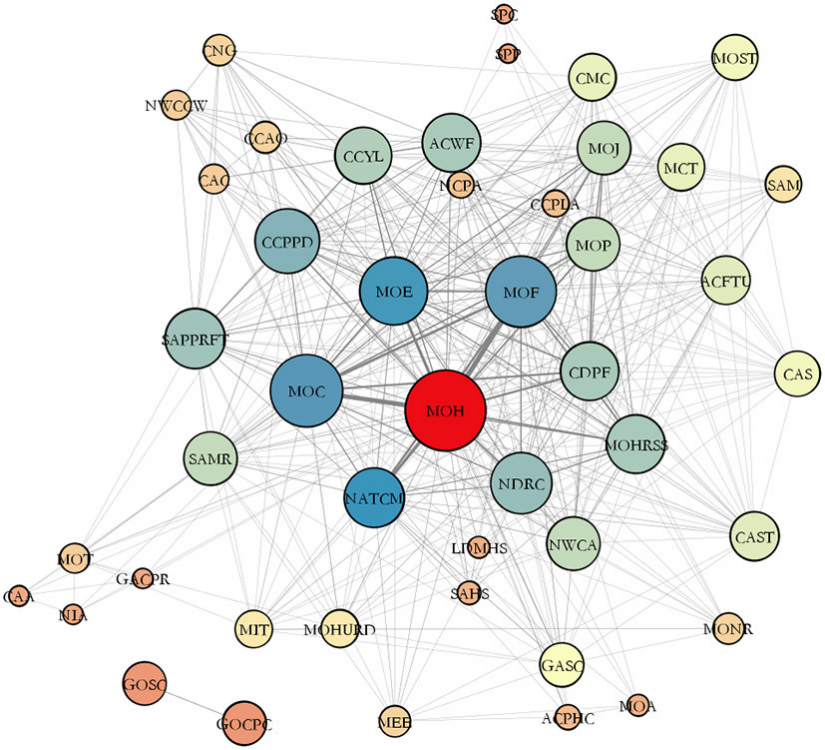
Network of mental health related policies making agencies in China.

Figure 6 shows a collaborative network of agencies involved in policy issuing, clarifying which departments are interconnected. The network structure presents a distinct edge-core layer.

The strengths of each agency and its synergies can be observed based on the size and location of the nodes. The MOH was at the center of the collaborative network. The MOF, the MOC, the MOE, and the National Administration of Traditional Chinese Medicine (NATCM) were the four nodes most closely linked to the MOH. However, the National Healthcare Security Administration (NHSA), which is responsible for promoting and implementing medical security policies for people with mental disorders, was at the edge of the collaborative network. The NDRC, the MOP, the CDPF, and the All-China Women's Federation (ACWF), which offer protection for mental health services for vulnerable groups, were in the middle level of the collaborative network. Agencies, such as the State Administration for Market Regulation (SAMR), China Cares for the Next Generation Working Committee (CNG), and the All-China Patriotic Health Campaign Committee (ACPHC) were on the edge of the synergistic network.

## Discussion

Our study indicated that the number of single annual issuances and joint interdepartmental issuances of mental health related policies was continuously increasing. Additionally, an increasing number of departments were involved in mental health, which is consistent with the research conclusions of Li et al. (37), implying that mental health issues were receiving greater attention from the Chinese government.

There may be three main reasons why mental health has received widespread attention. First, the seriousness of mental health problems has resulted in a heavy economic and social burden on the government, society, and families (38). The total annual per capita cost of schizophrenia was RMB 15,669.60 in some regions of China in 2004 and its direct cost accounted for 33.4% (39). Second, with the increasing number of common mental disorders, the demand for mental health services among the public is also rising (40). Notably, some studies have underlined that most people with mental disorders were not receiving appropriate mental health services (41). Third, mental and physical health interact and influence each other. Mental disorders could affect individuals' health behavior through the nervous system and indirectly result in social problems, such as suicide, violent crime, as well as drug and alcohol abuse (42). In the context of the problems mentioned above, the Chinese government has been gradually improving the mental health system.

We have found that the scope of service objects of mental health related policies is expanding, which is important for improving the equity and accessibility of mental health services (43). This finding was confirmed by a previous study by Jacka et al. (44) which noted an imbalance in service objects of mental health in Australia and called for a shift in emphasis toward prevention and promotion of the general population (44). In addition, more and more research has extended the service to a wider range of groups (45, 46). This phenomenon may be related to the following reasons. With the development of society, the population and characteristics of mental disorders are also changing (47). For example, in aging societies, the mental health problems of elderly people have become a common concern (48). Some studies have pointed out that people affected by major disasters and accidents need increased psychological assistance (49). Therefore, the Chinese government has formulated different mental health related policies for different specific groups of people, such as the elderly (50) and disaster-affected people (51).

However, the attention to resources in mental health related policies needs to be further improved, especially in terms of financial support. The possible reason for this phenomenon is as follows: the problem of inadequate financial funding for mental health services provided by governments has existed for a long time. Although the new health care reform policy has made it clear that the government should implement full subsidies for mental health institutions, there was a lack of clear and specific regulations regarding the level and direction of investment. It was reported that China's health department allocated only 2.3% of the financial allocation to psychiatric hospitals and 3.1% to other mental health related service institutions (52). This also reflected the conclusions of the previous studies that the lack of a unified financial plan and corresponding supporting measures resulted in limitations for the development of mental health care (53, 54).

Our study showed that the MOH was in a dominant position in the collaborative network of mental health related policies. This is consistent with the health sector playing a central role in the mental health service system in countries such as the USA and the UK (55, 56). It might be related to the functional attributes of the mental health system. The MOH includes prevention and treatment as well as rehabilitation and its functions cannot be implemented without the participation of medical institutions and community mental health centers. Therefore, the prevention and treatment of mental health problems have placed more emphasis on the primary responsibility of the MOH. In the future construction of the mental health system, the MOH should play its leading role and take the responsibilities of planning, organization, and implementation of mental health care policies.

Moreover, we found that the number of policies issued jointly was still fewer than the number of policies issued separately, indicating that collaboration among agencies still needs to be strengthened. Further, some agencies that should play a key role were on the edge of the collaborative network, such as the NHSA. A reason for this may be that the objects of medical insurance were mainly inclined toward disadvantaged people at the beginning of the system design, with limited coverage for people with common mental disorders. Currently, only a few regions in China have included common psychological treatments in the coverage of medical insurance (57), which also reflects the inadequacy of cross-sector collaboration. The implementation of mental health services involves various aspects of society and is influenced by background systems, culture, and other factors (58, 59). Therefore, it is difficult for it to be done by the MOH alone (60). Indeed, many other countries and regions have been facing similar challenges of inadequate collaboration among various departments in the field of mental health (61). For example, He et al. described the collaboration between child welfare and mental health agencies in Los Angeles County. The results highlight the significant role of intersectoral collaboration in facilitating mental health assessment of adolescents (62). Multisectoral collaboration has become an urgent global issue. In the future, the experiences of mental health governance in developed countries can be considered and promoting the implementation of mental health services through integrated community teams can be explored. Furthermore, effective cross-sectoral collaboration mechanisms can be established by integrating various resources (63).

Finally, it was significant that the number of mental health policies in 2020 showed a linear increase following the COVID-19 outbreak. Previous studies on outbreaks of infectious diseases and other catastrophic events have shown that these events have a devastating and lasting impact on mental health (64). It has been noted that the COVID-19 pandemic has impacted the mental health of the public, patients, medical personnel, children, and the elderly. Work stoppages and school suspensions caused by COVID-19 have been closely associated with increased psychological problems such as anxiety and depression (65, 66). Dembech et al. pointed out that it is necessary to strengthen the preparation of countries' health policies to ensure that they have sufficient capacity to cope with the potential impact of a pandemic (67). Therefore, the implications of COVID-19's effects on the population's mental health should be considered in future policy refinements.

## Limitations

There are several limitations of this study. First, although we have developed standard criteria for the content analysis of policies, researchers inevitably make judgments with some subjectivity, which may impact the results. Second, the policy documents can only represent a simple network of policy-making collaborations. The collaboration in policy-making among government agencies is highly complex and cannot be reflected only in the networks created through the quantitative analysis of policy documents. Further research could be combined with qualitative interviews of relevant experts and department heads for a more comprehensive analysis of the policies. Moreover, this study focused only on China. Further research could be conducted to compare policy changes in China with other countries and to talk about how contextual, cultural, and institutional can impact mental health policies.

## Conclusion

This study is the first attempt to comprehensively analyze the evolution of the dynamics of mental health related policies in China over the past 30 years through bibliometric analysis. This study can promote mental health service practices by domestic and international stakeholders and enrich policy research in the mental health area. Additionally, the results of this study can provide valuable insights for developing countries that have built mental health systems similar to China's.

We found that the policy content on mental health was enriching. However, cross-sector collaboration among departments needs to be enhanced and the attention to financial support also needs to be strengthened. Future policy design should focus on improving collaborative mechanisms in various mental health departments in China. It can be done by strengthening government leadership, refining the division of responsibilities among departments, and improving the system of penalties and incentives. Simultaneously, sustainable financing mechanism should be established by improving supervisory mechanism and developing diversified funding models.

## Data availability statement

The raw data supporting the conclusions of this article will be made available by the authors, without undue reservation.

## Author contributions

MH and CL conceptualized and designed this study. PS, QS, ZZ, ZC, CP, LX, ZH, AM, ZG, TX, PW, HW, and CH developed methodologies for data collection and analysis. HL, QZ, and HZ participated in data collection and analysis. HL and QZ participated in writing and editing. All authors contributed to the article and approved the submitted version.

## Funding

This study was funded by the Tsien Hsue-Shen Urbanology Award of Hangzhou International Urbanology Research Center and Zhejiang Urban Governance Studies Center (Grant number: 21QXS004), the National Natural Science Foundation of China (Grant numbers: 72074048 and 71774031), the Three-Year Action Plan of Shanghai Municipality Strengthens Public Health System Construction (Grant numbers: GWIV-32 and GWV-12), and the Shanghai Foundation for Talents Development (Grant number: 2020128).

## Conflict of interest

The authors declare that the research was conducted in the absence of any commercial or financial relationships that could be construed as a potential conflict of interest.

## Publisher's note

All claims expressed in this article are solely those of the authors and do not necessarily represent those of their affiliated organizations, or those of the publisher, the editors and the reviewers. Any product that may be evaluated in this article, or claim that may be made by its manufacturer, is not guaranteed or endorsed by the publisher.

## Abbreviations

MOH: Ministry of Health of the People's Republic of China
MOP: Ministry of Public Security of China
MOC: Ministry of Civil Affairs of the People's Republic of China
MOF: Ministry of Finance of China
MOE: Ministry of Education of China
CDPF: China Disabled Persons' Federation
NDRC: National Development and Reform Commission
SC: State Council of China
NHSA: National Healthcare Security Administration
NATCM: National Administration of Traditional Chinese Medicine
ACWF: All-China Women's Federation
SAMR: State Administration for Market Regulation
CNG: China Cares for the Next Generation Working Committee
ACPHC: All-China Patriotic Health Campaign Committee
MOHRSS: Ministry of Human Resources and Social Security of China
CCPPD: Publicity Department of the Communist Party of China
MOJ: Ministry of Justice
CCYL: The Central Committee of the Communist Young League
NRTA: National Radio and Television Administration
NWCA: National Working Commission on Aging
GJPC: Comprehensive Group of Joint Prevention and Control Mechanisms
NPCPRC: The National People's Congress of the People's Republic of China.
